# Experimental and Theoretical Studies on Enhanced Lubricity of Hyperbranched Polyamide-Amine for Water-Based Drilling Fluids

**DOI:** 10.3390/polym18131560

**Published:** 2026-06-23

**Authors:** Wei Wang, Rongsheng Lin, Lin Xu, Zhujun Zhang, Lei Wang, Siqi Yang, Wuwei Feng, Peng Xu, Meilan Huang

**Affiliations:** 1 School of Marine Engineering Equipment, Zhejiang Ocean University, Zhoushan 316022, Chinafengwuwei@163.com (W.F.); 2 Jurong Energy (Xinjiang) Co., Ltd., Urunqi 841603, China; linrongsheng@xjjurong.com; 3 CNOOC China Limited, Shanghai Branch, Shanghai 200330, China; 4 State Key Laboratory of Oil and Gas Equipment, CNPC Tubular Goods Research Institute, Xi’an 710076, China; 5 College of Petroleum Engineering, Yangtze University, Wuhan 430100, China; 6 School of Chemistry and Chemical Engineering, Queen’s University of Belfast, Belfast BT95AG, UK

**Keywords:** hyperbranched polymer, water-based drilling fluid, lubrication mechanism, molecular simulation, multi-site adsorption, tribofilm

## Abstract

High friction and drag are among the challenging subjects for constructing water-based drilling fluids available in horizontal drilling. Lubricants play a major role in mitigating friction of water-based drilling fluids, and thus, developing new lubricants is necessary for efficient horizontal drilling. In this work, a generation 1.5 (1.5G) hyperbranched polyamide-amine P(EDA-MA-OA), which serves as a candidate for a traditional lubricant with linear conformation, was newly synthesized via a divergent approach. A set of physicochemical characterizations was carried out on P(EDA-MA-OA) to confirm its effective synthesis. The results indicated that P(EDA-MA-OA) has a nanoparticulate morphology with a size of approximately 100 nm. Its molecular structure shows strong thermal stability, with initial thermal decomposition occurring at 146 °C. The water-based drilling fluid formulated with P(EDA-MA-OA) as the lubricant exhibits effective comprehensive properties and, in particular, the lubrication coefficient was 0.067, comparable to that of the oil-based drilling fluid, indicating enhanced lubricity by the incorporation of the hyperbranched polymer. The results of molecular simulations show that P(EDA-MA-OA) possesses a unique “basket-like” architecture, with C18 long chains enveloping the central active segments, namely the carbonyl (-C=O) and amide (-CO(NH_2_)) groups. When interacting with montmorillonite (MMT) particulates, the active groups can interact with MMT, allowing the eight C18 branched terminal chains to form a “molecular brush” with a normal orientation toward the MMT interface, which can serve as a hydrophobic lubricating film to improve lubricity. A lubrication model was finally proposed to rationalize the enhanced lubricity from the hyperbranched polymers in the water-based drilling fluid.

## 1. Introduction

With the widespread application of directional drilling technology in the petroleum industry, the role of drilling lubricants has become increasingly critical. Lubricants can not only mitigate friction and wear during directional drilling, but also contribute largely to minimizing drilling accidents. However, traditional lubricants such as base oils and chain polymers exhibit clear limitations in achieving efficient, long-term lubrication [1,2]. Therefore, the development of novel alternatives for water-based drilling fluids has become a key priority in oilfield chemistry.

Lubricants for water-based drilling fluids can be classified into different chemical categories, including vegetable oils, polyether alcohols, and alkyl polyglucosides. The polar groups, such as hydroxyl, amine, carboxyl, and thiol groups containing N, O, S, or P elements, can adsorb onto the friction pair surfaces through physical or chemical interactions, while the nonpolar long chains align directionally on the surfaces, forming a hydrophobic protective film during the lubrication process [3,4,5]. This strategy has been widely employed to modify lubricants, thereby enhancing their adsorption capacity and the strength of the lubricating film. Following this concept, researchers have designed and developed various polymeric lubricants and have introduced solid lubricants to achieve synergistic effects, obtaining good friction reduction and torque reduction performance [6,7,8,9]. Furthermore, to enhance the adsorption of molecules on friction surfaces and improve film-forming strength, researchers have explored ionic liquids as additives [10,11], which improve the lubricity and friction-reducing performance of water-based drilling fluids and show great potential. However, field data indicate that lubricating performance continuously declines as the drilling fluid circulates, which can be ascribed to damage to the molecular structure under high temperature or shear and failure of the surface film under high contact loads in confined spaces [12]. Therefore, improving the lubricity of water-based drilling fluids is focused on enhancing the adsorption stability of lubricant molecules with solid particles in the drilling fluid, which will be helpful to promote surface film formation. This necessitates a conformational change in the lubricant molecules. But the fact that conventional modification approaches change functional groups, monomers, or molecular weight appears to be limited in improving molecular adsorption. Consequently, designing unique molecular topological structures, e.g., comb-shaped, star-shaped, branched, and other three-dimensional conformations, to replace conventional linear structures can not only enable multiple-site adsorption for lubricant molecules, but also further enhance the shear resistance and thermal degradation resistance of the molecular structure [13,14,15,16]. This special conformation will significantly improve film-forming ability at the interface.

Hyperbranched polymers possess a radially symmetric, nanoscale architecture with a homogeneous and monodisperse structure. This well-defined core not only ensures structural uniformity but also leads to versatile functionalization, enabling precise tailoring of their physicochemical properties [17,18]. Due to the presence of numerous functional groups and compact molecular architecture, hyperbranched polymers demonstrate enhanced structural stability in harsh environments compared with traditional chain polymers [19,20]. Within oilfield chemistry, hyperbranched polymers have served as multifunctional additives in water-based drilling fluids, acting as viscosifiers, filtrate reducers, and inhibitors [21,22]. Recently, hyperbranched polymers as lubricants in cartilage-like composites within the biotribology system have been extensively reported [23,24,25,26]. The predominant lubrication theory reveals that the branched structure of aggrecan in synovial fluid forms a hydrophobic brush layer, which is crucial for surface lubrication and load bearing. Given their topological similarity to aggrecan, hyperbranched polymers can synergistically adsorb onto dispersed solid particulates in drilling fluids, forming a protective interface to effectively mitigate drilling friction and wear. Despite their broader potential in drilling fluids, investigations into hyperbranched polymers as lubricants appear to be limited. Zhong [27] and Wang [28] analyzed the structural characteristics and size effects of hyperbranched macromolecules, highlighting that molecular topology-based architectural design represents a crucial strategy for developing high-performance drilling fluid additives. Yan et al. [29] synthesized a bio-lubricant XZ-RHJ, which contains multiple strongly adsorbing functional groups and is capable of forming a shear-resistant film on both the wellbore and the drill string, effectively reducing frictional resistance. Jia et al. [30] reported a branched lubricant synthesized from long-chain fatty acids and polyols. Its multiple polar adsorption groups are capable of forming multiple hydrogen bonds with rock and metal surfaces, thereby reducing the lubrication coefficient of the drilling fluid by over 90%. Gao et al. [31] designed and synthesized an acrylate-based polymer brush, in which the densely packed, long side chains terminated with hydroxyl groups significantly enhanced the strength of the lubricating film, leading to a 91.2% reduction in the friction coefficient of the drilling fluid.

Inspired by the enhanced lubricity of hyperbranched macromolecules in the biotribology system, in this work, a novel 1.5th generation (1.5G) hyperbranched polymer, P(EDA-MA-OA), was designed and synthesized using ethylenediamine (EDA), methyl acrylate (MA), and octadecyl acrylate (OA) through a divergent strategy. The corresponding chemical structure, thermal stability, and properties of the drilling fluid containing P(EDA-MA-OA) were comprehensively explored. Moreover, a structure–activity relation model of the hyperbranched polymer was proposed to elucidate its distinctive capacity for improving the lubricity of water-based drilling fluids. This research identified the hyperbranched polymer as a lubricant candidate for constructing new water-based drilling fluids.

## 2. Experimental Sections

### 2.1. Materials

EDA, MA, OA, and methanol were purchased from Shanghai Aladdin Biochemical Technology Co., Ltd., (Shanghai, China). Na_2_CO_3_, NaOH, and KCl were purchased from Shanghai Macklin Biochemical Technology Co., Ltd., (Shanghai, China). Bentonite, filtrate reducer DFD, viscosifier VIS, coating agent PLUS, and lubricant Lube were supplied by Jingzhou Jiahua Technology Co., Ltd., (Jingzhou, China).

### 2.2. Synthesis of Hyperbranched Polyamide-Amine

**Figure 1:**
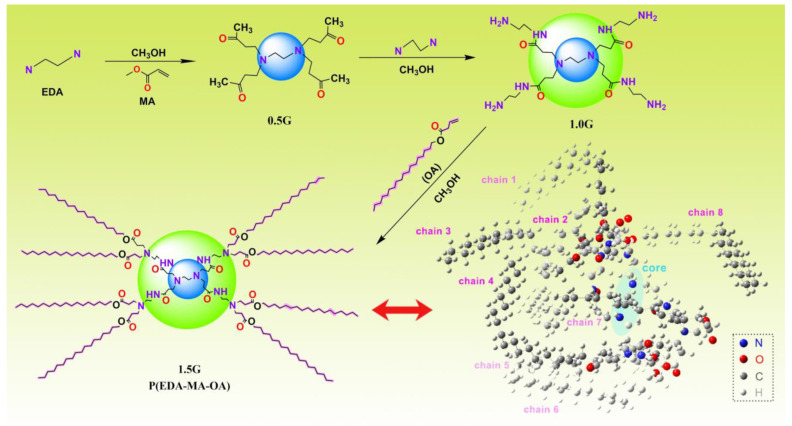
Synthetic route of 1.5G P(EDA-MA-OA). The blue and green circular areas correspond to 0.5G and 1.0G, respectively.

The 1.5G polyamide-amine hyperbranched polymer, P(EDA-MA-OA), was synthesized via a divergent approach, and the synthetic process is shown in Figure 1. Firstly, 0.5G P(EDA-MA-OA) was synthesized via a Michael reaction of EDA with MA, wherein EDA served as a quadrivalent core, and MA worked as a building monomer. A total of 18 g (0.3 mol) EDA and 60 mL methanol were added into a three-neck flask to prepare the EDA solution, and then 206.4 g (2.4 mol) MA was dropped into the EDA solution at a rate of 2 drop/sec at a stirring speed of 300 r/min in the ice-water bath. After the drop, the Michael reaction was maintained for 24 h, and, in the meantime, N_2_ was continuously injected. The product 0.5G P(EDA-MA-OA) was handled with vacuum distillation at 45 °C for purification. Secondly, 1.0G P(EDA-MA-OA) was further synthesized with 0.5G P(EDA-MA-OA) and MA via an aminolysis reaction. In total, 40.4 g (0.1 mol) 0.5G P(EDA-MA-OA) and 120 mL methanol were mixed in the three-neck flask, and then 144 g (2.4 mol) EDA was dropped at 1 drop/sec at 300 r/min. After the drop, the aminolysis reaction and N_2_ injection were maintained for 24 h. Similarly, the obtained product 1.0G P(EDA-MA-OA) was handled with vacuum distillation at 45 °C. Finally, 1.5G P(EDA-MA-OA) was tailored via another Michael reaction of 1.0G P(EDA-MA-OA) with OA, wherein OA served as a terminated monomer. A total of 100.32 g (0.02 mol) 1.0G P(EDA-MA-OA) and 30 mL methanol were mixed. Then, 77.76 g (0.24 mol) OA was added to 90 mL methanol, and, subsequently, the OA solution was dropped at 1 drop/sec at 300 r/min in the ice-water bath. Meanwhile, the reaction and N_2_ injection were maintained for 24 h. The obtained product was purified by vacuum distillation at 45 °C.

### 2.3. Characterization of Hyperbranched Polyamide-Amine

The physicochemical characterization of 1.5G P(EDA-MA-OA) was conducted via Fourier transform infrared spectroscopy (FTIR), nuclear magnetic resonance hydrogen and carbon spectroscopy (^1^H- and ^13^C-NMR), thermogravimetric analysis (TGA), and transmission electron microscopy (TEM). Fourier transform infrared (FTIR) spectra were recorded on a spectrometer (Nicolet iS50, Thermo Fisher Scientific Inc., Waltham, MA, USA) in the range of 4000–400 cm^−1^ with a resolution of 4 cm^−1^. A total of 32 scans were accumulated for each spectrum, and the scanning speed of the interferometer mirror was set to 2 mm/s. The NMR spectra were acquired on an Advance III HD 400 liquid NMR spectrometer (Bruker Corporation, Billerica, MA, USA). The sample was dissolved in CDCl_3_ (ca. 15 mg/mL) with TMS as the internal standard (δ = 0 ppm). ^1^H- and ^13^C-NMR spectra were recorded on a 400 MHz spectrometer. For ^1^H-NMR, 32 scans were acquired with a relaxation delay of 2 s and a 30° pulse angle. For ^13^C-NMR, 2048 scans were accumulated with broadband proton decoupling and a relaxation delay of 2 s. The thermal stability was determined on a TGA 550 thermogravimetric analyzer (TA Instruments, New Castle, DE, USA) within a temperature range of 20–800 °C at a heating rate of 10 °C/min. The morphology and dimensions of the product were examined using a Hitachi HT7800 TEM (Hitachi High-Tech Corporation, Tokyo, Japan).

### 2.4. Preparation of Water-Based Drilling Fluid

Based on the formula, a low-solid, freshwater-based drilling fluid was prepared: 2 wt.% bentonite + 0.25 wt.% pH-adjusting agent (0.15 wt.% Na_2_CO_3_ + 0.1 wt.% NaOH) + 2 wt.% filtrate reducer DFD + 0.15 wt.% viscosifier VIS + 4 wt.% inhibitor KCl + 0.3 wt.% coating agent PLUS + 2 wt.% lubricant. In this case, P(EDA-MA-OA) and Lube were utilized as lubricants to compare their lubrication performance, wherein Lube is a linear ester molecule and is typically utilized as the lubricant on-site. The thermal aging tests of the drilling fluids were performed in a GRL-9 roller at 120 °C for 16 h. It is noted that the aging temperature of water-based drilling fluid investigated here was determined on the basis of the formation conditions of the eastern Sichuan shale gas field, and all functional additives mentioned here were commercially available. For simplicity, the term 1.5G P(EDA-MA-OA) was replaced by P(EDA-MA-OA) in the following sections, unless otherwise specified.

### 2.5. Tests of Water-Based Drilling Fluid Properties

The rheological characteristics such as apparent viscosity (AV), plastic viscosity (PV), and yield point (YP) were examined using a rotating viscometer. Filtration loss (FL) assays were conducted on a medium-pressure filtration device. Lubricity evaluations were executed via an extreme pressure lubricity tester (EP 212, Hangzhou Chincan Trading Co., Ltd., Hangzhou, China). All tests of drilling fluid properties were carried out in accordance with the American Petroleum Institute specification [14]. All measurements were performed three times, and the results are reported as means and standard deviations. It should be pointed out that the objective of this work was to develop a hyperbranched polymer-based water-based drilling fluid capable of replacing the oil-based drilling fluid currently used in the eastern Sichuan shale gas field. Hence, after verifying that the basic properties (rheology, filtration, etc.) of the formulated water-based fluid met the required standards, the lubricity of the two fluids was compared under identical conditions.

### 2.6. Tests of P(EDA-MA-OA)-Bentonite Architecture

To investigate the film-forming behavior of the lubricant on the surfaces of the friction pair, the microstructure and surface elemental compositions of the dried filter cake samples from the lubricating base mud were comparatively examined using SEM and EDS (SU8010, Hitachi High-Tech Corporation, Tokyo, Japan). To minimize the influence of other additives, the lubricating base mud consisted only of the lubricant and a pH regulator, with the specific formulation as follows: 2 wt.% bentonite + 0.25 wt.% pH-adjusting agent (0.15 wt.% Na_2_CO_3_ + 0.1 wt.% NaOH) + 2 wt.% lubricant.

### 2.7. Molecular Simulation

#### 2.7.1. Construction of Molecular Models

The molecular model was constructed using the Materials Studio 2019 software package to build a hyperbranched polymer model. The modeling steps were as follows: designing an ethylenediamine core, using acrylamide as the chain-extending unit, and acrylate octadecyl as the end-capping monomer to construct P(EDA-MA-OA). In the Forcite module, the COMPASS II force field [32] was selected to perform structural optimization on the P (EDA-MA-OA) molecular model. The iteration step was set to 5000, and the energy was converged to 1.0 × 10^−4^ kcal/mol, resulting in a stable molecular configuration.

The clay phase is primarily composed of montmorillonite (MMT). Based on the MMT unit cell model parameters [33], a molecular model of the clay phase was constructed using Materials Studio 2019. The (0 0 1) crystal plane of montmorillonite is the most stable in the reservoir [34], and this plane was designated as the adsorption surface of the clay phase. Similarly, in the Forcite module, the COMPASS II force field was selected to perform energy minimization for the MMT molecular model.

#### 2.7.2. Approaches of Molecular Simulation

Quantum chemical calculations were performed on P (EDA-MA-OA) to determine the active site and its distribution. In the Dmol3 module, molecular structure optimization and density functional theory (DFT) calculations were conducted on P (EDA-MA-OA). Specifically, the generalized gradient approximation (GGA)/PBE functional method [35] was employed for geometric optimization of P (EDA-MA-OA). A two-valence polarization basis set was used, with a self-consistent field convergence threshold of 2.0 × 10^−6^ eV/atom to ensure the molecular structure reached the lowest-energy stable state. Electron density and distribution of P (EDA-MA-OA) were calculated, followed by electrostatic potential (ESP) and frontier molecular orbital (HMO) analyses to define active atoms and obtain distribution characteristics.

Molecular dynamics simulations were conducted on the P(EDA-MA-OA)-MMT composite system to investigate the influence of hyperbranched polymers on interfacial adsorption states through variations in molecular size, active atom distribution, and adsorption energy. Using the Forcite module, equilibrium simulations were first performed in the NVT ensemble with Nose temperature control, covering a temperature range of 278 K to 513 K. Van der Waals and electrostatic interactions were modeled using the atom-based Ewald addition method and the truncated-radius method, respectively, with a truncated radius of 1.55 nm. Molecular dynamics simulations were then carried out at 298 K with a time step of 1 fs, totaling 60 ns. Data from the final 10 ns were analyzed to ensure the system reached equilibrium. After geometric optimization of the composite system with fixed MMT surface atoms, adsorption simulations were performed in the NVT ensemble to analyze the adsorption configurations and trends in adsorption energy of PAMAM molecules on the interface.

The molecular conformation of P(EDA-MA-OA) in the equilibrium state of the composite system was selected. Using the Multiwfn 3.8 program, an independent gradient model method based on Hirshfeld partitioning [36] was employed to calculate the interatomic interaction region defined by the three-dimensional function. The VMD 2.0 software was utilized to visualize the short-range van der Waals forces between carbon chain segments, further elucidating the spatial arrangement of the hyperbranched polymer terminal chains in the P(EDA-MA-OA)-MMT composite system. It should be pointed out that the molecular simulations in this work were performed under simplified conditions but can provide valuable information on the multi-site adsorption and spatial conformation of the hyperbranched polymer.

## 3. Results and Discussion

### 3.1. FTIR Analysis

**Figure 2:**
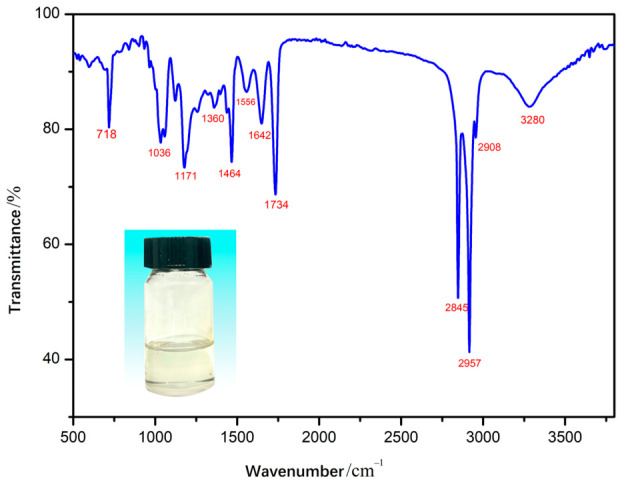
FTIR spectra of 1.5G P(EDA-MA-OA).

Figure 2 presents the FTIR spectra of the synthesized hyperbranched polymer P(EDA-MA-OA). The -CONH- group was represented by characteristic peaks at 3280, 1556, and 1642 cm^−1^. Specifically, the peaks at 3280 and 1556 cm^−1^ were ascribed to the stretching and in-plane bending vibrations of N-H, respectively, while the peak at 1642 cm^−1^ arose from the C=O stretching vibration. The C=O stretching vibration in the ester group was responsible for the peak at 1733 cm^−1^, and the peak at 1172 cm^−1^ was associated with the ester group. The -C-N- stretching vibration corresponded to the peak at 1036 cm^−1^. Strong peaks were observed at 2908, 2957, and 2845 cm^−1^, which were related to asymmetric -CH_2_ groups, implying a high molecular ratio in P(EDA-MA-OA). Notably, the out-of-plane bending vibration of -CH_2_- at 718 cm^−1^ emerged, corresponding to the molecular fragment -C-(CH_2_)n-C-(n ≥ 4), demonstrating that the end monomer OA with a long chain -(CH_2_)_17_- was successfully incorporated into the hyperbranched polymer.

### 3.2. NMR Analysis

**Figure 3:**
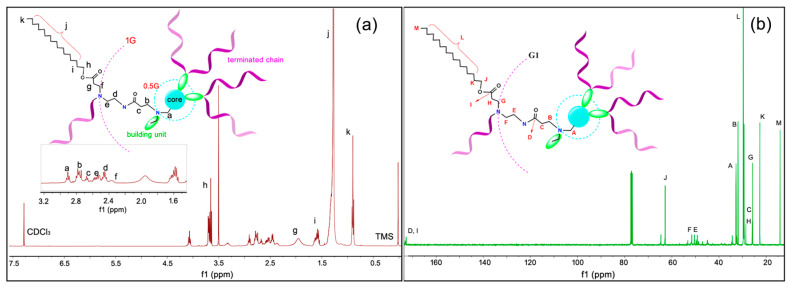
NMR spectra of P(EDA-MA-OA): (**a**) ^1^H-NMR, (**b**) ^13^C-NMR.

Figure 3 displays the ^1^H-NMR and ^13^C-NMR spectra of the synthesized P(EDA-MA-OA) product. The 1H-NMR spectrum distinctly showed the characteristic signals of the target structure. The core methylene protons (-CH_2_-) were represented by the peak at 2.91 ppm. After the Michael addition, protons of the -CH_2_-CH_2_-CO- fragment produced signals at 2.80 and 2.68 ppm. Then, the amination reaction brought about the -N-CH_2_-CH_2_-N- fragment, as indicated by peaks at 2.46 and 2.55 ppm. Another Michael addition step generated additional -CH_2_-CH_2_-CO- signals at 2.37 and 1.95 ppm. The peaks at 3.66, 1.58, 1.32, and 0.90 ppm were ascribed to the methylene and terminal methyl protons from the octadecyl chains introduced by OA, with the strong signal at 1.32 ppm signifying the repeating -(CH_2_)_17_- fragment. Solvent and calibration peaks were seen at 0.03 ppm (TMS), 7.28 ppm (CDC_l3_), and 3.53 ppm (residual methanol). The presence of methanol in the NMR sample implied that the hyperbranched structure of P(EDA-MA-OA) could encapsulate solvent molecules. Together with the FTIR results, these NMR data verified the successful synthesis of the target hyperbranched polymer with eight long alkyl chains.

The ^13^C-NMR spectrum of P(EDA-MA-OA) offered additional structural validation. Notable signals featured those at 32.8 ppm (core methylene carbon, -CH_2_-), along with those at 32.9 ppm and 25.9 ppm, which corresponded to the methylene carbons of the extended acrylate fragment (-COO-C-CH_2_-). The most prominent signal at 29.6 ppm was typical of the repeating methylene units in the long chain (-(CH_2_)_17_-). Importantly, the distinctive peak at 173 ppm was attributed to the carbonyl carbon of the -NCO- group, serving as a direct indicator of the consecutive Michael addition and amination reactions. These ^13^C-NMR findings jointly verified the anticipated molecular structure of P(EDA-MA-OA).

### 3.3. TEM Analysis

**Figure 4:**
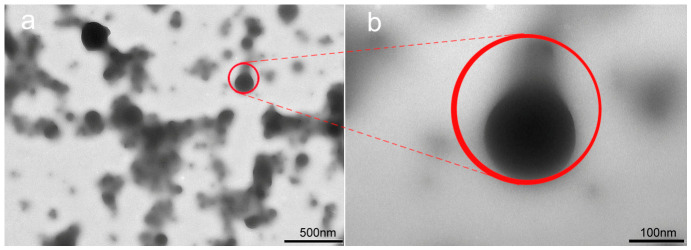
TEM image of (**a**) the aggregated state and (**b**) the individual for P(EDA-MA-OA).

Figure 4 presents the morphology of the synthesized P(EDA-MA-OA) in detail. It can be clearly seen in Figure 3a that the hyperbranched polymers are in an aggregated state. Figure 3b shows a magnified view of the red area marked in Figure 3a, demonstrating that individual hyperbranched polymer particles are spherical with a size of 100 nm. Apparently, These particles could be accurately characterized as discrete nanoaggregates. It can be reasonably concluded that such a compact spatial morphology should contribute largely to both structural stability and thermal resistance [37].

### 3.4. Thermal Property

**Figure 5:**
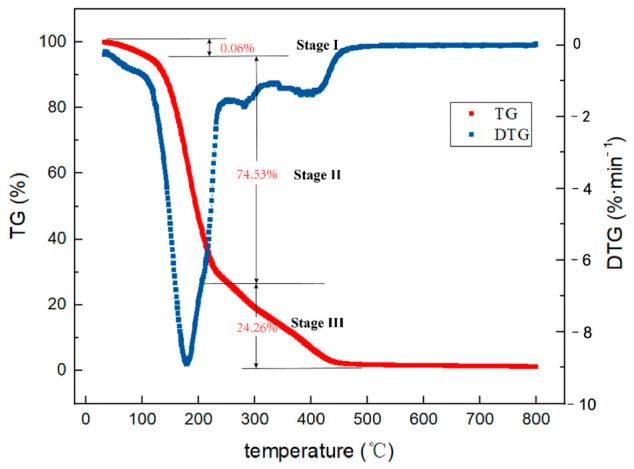
Pyrolysis curve of P(EDAMA-OA).

To further quantify the thermal stability, the initial decomposition temperature, maximum decomposition temperature, and residual mass at 800 °C were calculated from the TG-DTG curves (see Figure 5). The initial decomposition temperature was determined to be approximately 146 °C, and the maximum decomposition temperature for the second stage was found at 214 °C, as indicated by the peak in the DTG curve. For the third stage, a relatively lower decomposition was observed at 389 °C, reflecting the slower decomposition of the more thermally robust core. The residual mass remaining at 800 °C was 1.15%, which should correspond to the formation of inorganic carbonaceous residues from the incomplete combustion of the polymer backbone. These comprehensive thermal analyses not only confirmed the structural integrity of P(EDA-MA-OA) under elevated temperatures but also provided critical insights into its thermal degradation mechanism, which is essential for predicting its performance and service life upon drilling.

### 3.5. Properties of Water-Based Drilling Fluids

**Figure 6:**
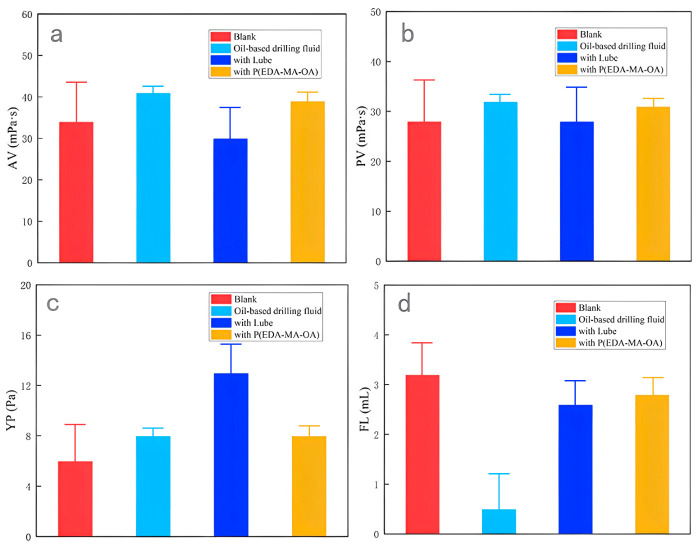
Comparison of rheological and filtration properties for drilling fluids referred to here: (**a**) AV, (**b**) PV, (**c**) YP, and (**d**) FL.

To assess its practical effectiveness, P(EDA-MA-OA) was introduced as a lubricant additive in water-based drilling fluid. A standard polymer/KCl system [38] served as the base fluid for comparison, favoring an aqueous option over conventional oil-based drilling fluids [39]. The rheological and filtration characteristics of drillings with and without P(EDA-MA-OA) were comparatively examined, with the results presented in Figure 6. Incorporation of P(EDA-MA-OA) led to an acceptable AV of 39 mPa·s and a low FL of 2.8 mL, which were similar to those of the polymer/KCl system, showing the ability of P(EDA-MA-OA) to adjust drilling fluid properties. Moreover, the drilling fluid containing P(EDA-MA-OA) exhibited a secondary rolling recovery rate surpassing 90%, proving outstanding clay inhibition. All these findings verified the potential of P(EDA-MA-OA) for the construction of novel lubricating drilling fluids.

### 3.6. Lubricity of Water-Based Drilling Fluids

**Figure 7:**
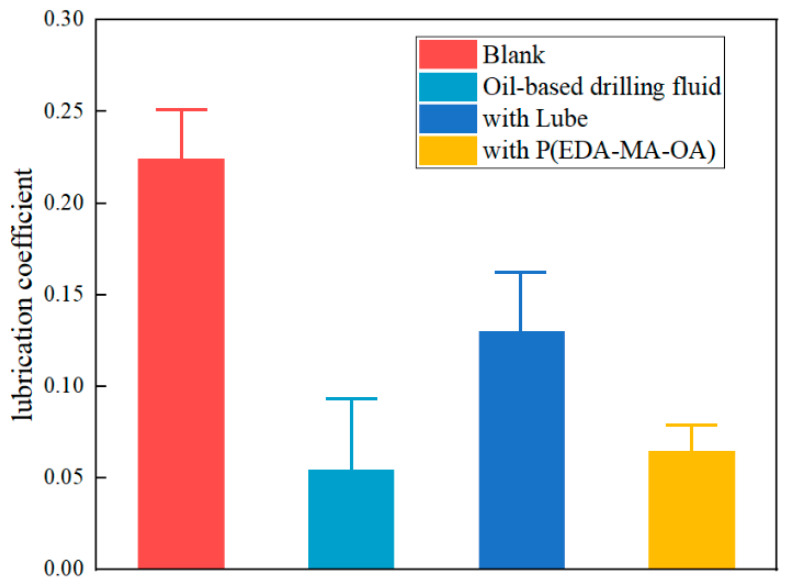
Comparison of lubrication coefficient of different drilling fluids.

The lubricity of drilling fluids was quantitatively evaluated by comparing the lubrication coefficients in Figure 7. The coefficient varied dramatically from 0.231 to 0.068 upon adding P(EDA-MA-OA), decreasing by 70.6%, which was comparable to that of conventional oil-based drilling fluid. This significant reduction confirmed the superior lubrication performance of the P(EDA-MA-OA)-containing drilling fluid. This result demonstrated that the hyperbranched polymer P(EDA-MA-OA) can effectively enhance the lubricity of water-based systems to a level approaching that of oil-based drilling fluids.

The preceding results indicated that the introduction of the hyperbranched polymer can significantly enhance the lubricating performance of the corresponding water-based drilling fluid. This enhancement should be closely related to the spatial configuration of the hyperbranched polymer and its microscopic interactions with the drilling fluid system. Therefore, further molecular simulation studies were performed on the spatial configuration, distribution of active atoms, and adsorption kinetics of the hyperbranched polymer P(EDA-AM-OA) onto clay particles, which would help elucidate the lubrication mechanism of the hyperbranched polymer.

### 3.7. Microstructure of P(EDA-MA-OA)-Bentonite Composite

**Figure 8:**
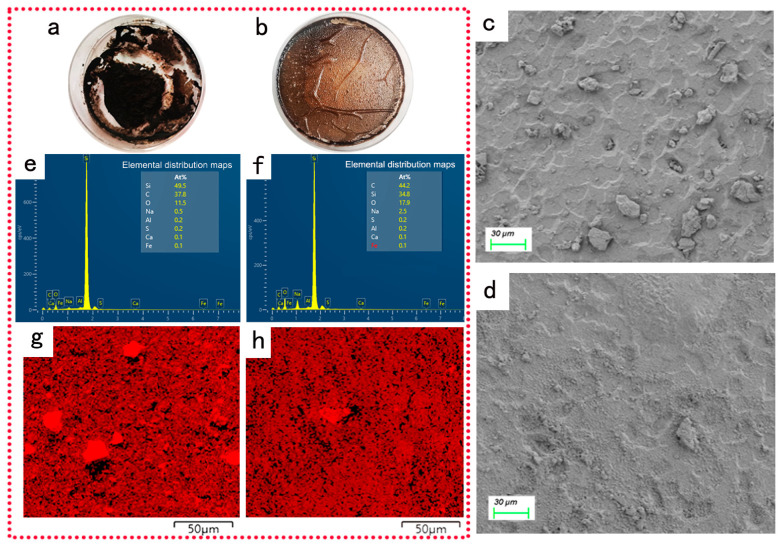
Comparison of lubricating mud cakes: (**a**) Lube-mud cake, (**b**) P(EDA-MA-OA)-mud cake, (**c**) SEM images of Lube-mud cake, (**d**) SEM images of P(EDA-MA-OA)-mud cake, (**e**) elemental distribution maps of Lube-mud cake, (**f**) elemental distribution maps of P(EDA-MA-OA)-mud cake, (**g**) C-distribution of Lube-mud cake, and (**h**) C-distribution of P(EDA-MA-OA)-mud cake.

As shown in Figure 8a,b, the lubricating base mud containing P(EDA-MA-OA) formed a dense and ductile film on the glass (Si-O(H)) surface, whereas the Lube-mud sample exhibited localized fracture and curling, failing to effectively cover the surface. These findings indicate that the hyperbranched polymer structure can not only enhance the adhesion of the film to the Si-O(H) surface, but also significantly improve the mechanical properties of the formed film. In Figure 8c,d, the Lube-mud sample surface appears to be rough, with numerous “island-like” coarse particles, suggesting uneven self-assembly and growth of bentonite particles. In contrast, the P(EDA-MA-OA)-mud sample shows a smooth surface, further demonstrating that the addition of the hyperbranched polymer improves the self-aggregation behavior of bentonite particles. This improvement is likely attributable to the multi-site adsorption of active atoms, which improves the arrangement of bentonite particulates, while the C18 terminal chains reduce surface tension, preventing the agglomeration of micron-sized bentonite particles and resulting in a more uniform surface morphology [40,41]. The interface layer of P(EDA-MA-OA) is relatively smooth and nanostructured on micrometric scales. This suggests that the polymer molecules are attached to the surface via multiple attachment sites.

Figure 8e,h present the elemental composition of the base slurry surface before and after the introduction of P(EDA-MA-OA). As expected, the contents of carbon (C) and oxygen (O) on the sample surface increased significantly with the addition of the hyperbranched polymer. In particular, the carbon content increased by approximately 7%, mainly originating from the hydrophobic terminal groups -(CH_2_)_17_-CH_3_ of P(EDA-MA-OA). Comparing the carbon elemental mapping of the two samples (Figure 8g,h), the distribution of carbon on the lubricated slurry surface is finer and denser than that on the blank sample surface, indicating that the hyperbranched polymer structure can uniformly spread and cover the bentonite matrix surface. These results demonstrate that the hyperbranched polymer P(EDA-MA-OA) can enhance the self-adsorption onto bentonite layers by virtue of its unique spatial molecular configuration. As a result of reinforced adsorption, a uniform and dense film can be formed. Undoubtedly, the hierarchical conformation of the hyperbranched polymer should play an important role.

### 3.8. Active Sites and Distribution of P(EDA-MA-OA)

The molecular surface electrostatic potential, *V*(*r*), serves as a powerful descriptor for mapping the spatial locations of active sites. *V*(*r*) represents the electrostatic potential generated by the nuclei and electrons of a molecule at any given point *r*, and its exact formulation is given by [42]:

(1) $$V(r)=\sum_{A}\frac{Z_{A}}{\left|R_{A}-r\right|}\;-\;\int \frac{\rho (r\prime )dr\prime }{\left|r\prime -r\right|}$$

where *Z_A_* and *ρ*(*r*) denote the charge of nucleus *A* at *R_A_* and the molecular electron density, respectively. The sign of *V*(*r*) in any region reflects the competition between positive nuclear and negative electronic contributions, thereby enabling the identification of active atoms and their spatial distribution in P(EDA-MA-OA).

**Figure 9:**
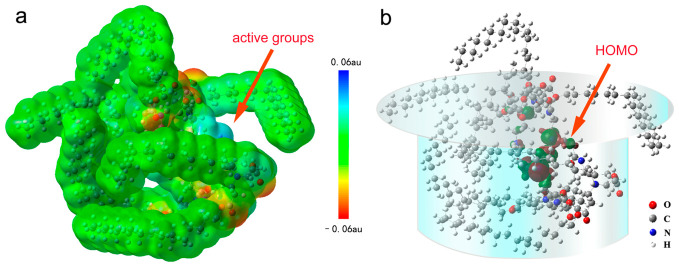
Description of active sites for P(EDA-MA-OA): (**a**) ESP, (**b**) HOMO.

Figure 9 presents the surface electrostatic potential and the highest occupied molecular orbital (HOMO) of the hyperbranched polymer. P(EDA-MA-OA) exhibits a basket-like configuration with a central cavity surrounded by long branched chains, which facilitates the extension of the flexible long chains. In the ESP map, the red regions correspond to a high proportion of lone pair electrons, indicating a strong tendency to donate electrons to the empty orbitals of ligand atoms. Accordingly, these regions correspond to higher local activity. Evidently, the active regions of P(EDA-MA-OA) are mainly concentrated on the central segments with the carbonyl groups such as -C=O and -CO(NH_2_). In addition, HOMO is localized on the central carbonyl group, further validating the active central carbonyl group. These active sites can interact with Si-OH groups on the bentonite interface, leading to the formation of P(EDA-MA-OA)-clay composites [43,44].

### 3.9. Adsorption of P(EDA-MA-OA) on the MMT Interface

**Figure 10:**
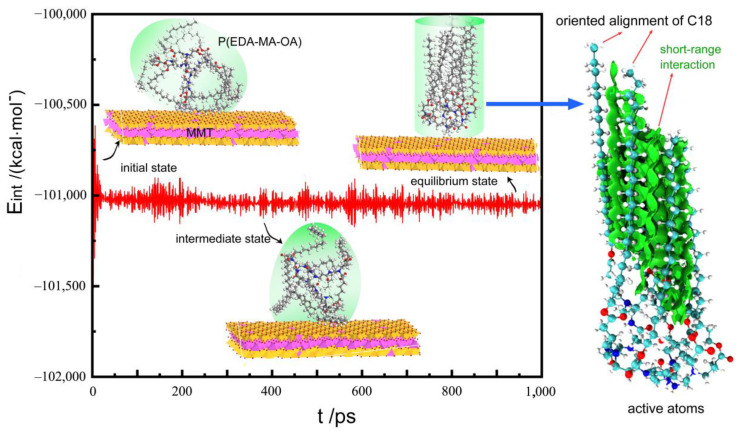
Adsorption kinetic situation of P(EDA-MA-OA) on the MMT surface.

Figure 10 presents the adsorption kinetics curve of P(EDA-MA-OA) on the montmorillonite (MMT) interface. The structural evolution of the P(EDA-MA-OA)-MMT composite undergoes three stages: initial, flipping, and vertical orientation. In the initial stage, the P(EDA-MA-OA)-MMT configuration is unstable, exhibiting large energy fluctuations, with an average energy of −100,756.88 kcal/mol. After 500 ps, a conformational flipping of the P(EDA-MA-OA) molecule occurs. In this case, the entire molecule shifts towards the MMT interface, due to the adsorption of the central active groups (-C=O and -CO(NH_2_)), while the outer C18 branched chains start to extend along the normal direction. After 900 ps, the steady conformation of the P(EDA-MA-OA)-MMT complex will be obtained, wherein P(EDA-MA-OA) exhibits a columnar extension perpendicular to the MMT interface. This conformation is characterized by (i) the central active segments with carbonyl groups (e.g., C=O and -CO(NH_2_)) prompting P(EDA-MA-OA) adsorption with MMT, and (ii) the eight C18 branched terminal chains forming a “molecular brush” with a normal orientation toward the MMT interface [45,46].

Figure 10 further illustrates the distribution of weak interactions among the C18 branched long chains. The green flake-like regions represent short-range van der Waals forces between adjacent long chains. This cohesive force ensures close packing of the long chains, leading to the formation of a hydrophobic film on the MMT interface, which greatly improves the solid lubricity of the composite.

### 3.10. Lubrication Model of Hyperbranched Polymer Water-Based Drilling Fluid

**Figure 11:**
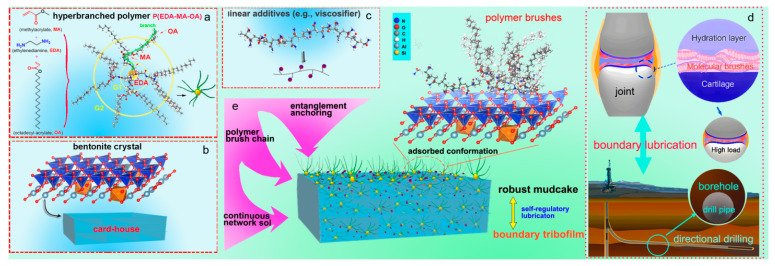
(**a**–**e**) The lubrication model of drilling fluids formulated with hyperbranched polymer: (**a**) P(EDA-MA-OA) configuration, (**b**) bentonite hydration, (**c**) linear additives, (**d**) boundary lubrication of joint and directional drilling.

The lubricating behavior of water-based drilling fluids formulated with the hyperbranched polymer is a multi-step process, as shown in Figure 11. The unique hyperbranched architecture of the polymer plays a crucial role by providing multiple adsorption sites, enabling the polymer to anchor firmly onto bentonite layers [47]. This firm anchoring induces the hydrophobic chains, specifically the -(CH_2_)_17_- segments, to organize into a brush-like surface layer [48]. Consequently, the polymer significantly improves the mud cake structure during the drilling process.

On the basis of the organization described above, the hyperbranched polymer exhibits a special spatial configuration, which can lead to a unique P(EDA-MA-OA)-MMT composite by means of interfacial adsorption. The hydrophobic film will be formed to reinforce the mud cake, thereby increasing its lubricating and load-bearing capacity. Consequently, a stable boundary lubrication layer is established, as observed in Figure 11. The robust mud cake can act as a sacrificial tribofilm, protecting the friction pair of the drilling tool from wear and friction [49]. In this regard, direct contact friction between the drilling tools and the rock is significantly reduced. The transition from severe boundary friction to milder film wear effectively mitigates drilling friction, thus enhancing the overall lubrication of the drilling process.

## 4. Conclusions

In conclusion, a novel hyperbranched polymer lubricant, P(EDA-MA-OA), has been successfully synthesized and characterized. The hyperbranched polymers have a nanosized spatial architecture and exhibited favorable thermal stability, which can facilitate the arrangement of bentonite particulates. The water-based drilling fluid with P(EDA-MA-OA) as the lubricant was constructed, which exhibits excellent lubricity, parallel to that of traditional oil-based drilling fluids. P(EDA-MA-OA) appears to be a “basket-like” spatial conformation, and the central active segments with carbonyl groups are surrounded by eight C18 terminal chains. These active segments can associate with the bentonite layer by means of weak interactions, and C18 terminal chains form a “molecular brush” with a normal orientation toward the bentonite interface, leading to the formation of a hydrophobic film. A novel boundary lubrication model was proposed to rationalize the outstanding lubricity, wherein the hyperbranched architecture can facilitate the formation of a mud cake as a sacrificial tribofilm.

This research established hyperbranched polymers as a promising candidate for the construction of highly lubricating drilling fluids. Future research will focus mainly on field application of the hyperbranched polymer-based drilling fluids and theoretical investigation into the structure–activity relationship of hyperbranched polymers in water-based drilling fluids.

## Data Availability

The data presented in this study are available on request from the corresponding author. (The data are not publicly available due to privacy or ethical restrictions.)

## Abbreviations

The following abbreviations are used in this manuscript:

P(EDA-MA-OA)	Generation 1.5 hyperbranched polyamide-amine
EDA	Ethylenediamine
MA	Methyl acrylate
OA	Octadecyl acrylate
FTIR	Fourier transform infrared spectroscopy
NMR	Nuclear magnetic resonance
TGA	Thermogravimetric analysis
TEM	Transmission electron microscopy
AV	Apparent viscosity
PV	Plastic viscosity
YP	Yield point
FL	Filtration loss
MMT	Montmorillonite
DFT	Density functional theory
ESP	Electrostatic potential
GGA	Generalized gradient approximation
PBE	Perdew–Burke–Ernzerhof functional
HMO	Frontier molecular orbital
HOMO	Highest occupied molecular orbital
*V*(*r*)	Molecular surface electrostatic potential
DFD\VIS\PLUS\Lube	Code of drilling fluid additive
